# Thirty years of NCI thyroid cancer research investment (1996–2025): a longitudinal portfolio analysis of funding trends, molecular target emphasis and thematic evolution

**DOI:** 10.3389/fonc.2026.1876183

**Published:** 2026-08-17

**Authors:** Youfang Jiang, Yaoyao Chen, Xinrong Lai, Fei Le

**Affiliations:** 1 Department of Head and Neck Surgery, Jiangxi Cancer Hospital, The Second Affiliated Hospital of Nanchang Medical College, Jiangxi Clinical Research Center for Cancer, Nanchang, China; 2 School of Clinical Medicine, Nanchang Medical College, Nanchang, China

**Keywords:** BERTopic, MedCPT, National Cancer Institute, research policy, thyroid cancer

## Abstract

**Background:**

Thyroid cancer research has shifted over the past three decades toward molecular classification, precision diagnostics and targeted therapy. However, the evolution of public research investment in this field has not been systematically characterized. This study analyzed National Cancer Institute (NCI)-administered thyroid cancer research funding from 1996 to 2025.

**Methods:**

We performed a longitudinal portfolio analysis of NIH RePORTER records from fiscal years 1996–2025. NCI-administered projects with explicit thyroid cancer research relevance were identified through review of project titles, abstracts, public health relevance statements and project terms. The final analytic cohort comprised 753 project-award years (AYs). Rule-based dictionaries were used to annotate research stage, disease subtype, molecular target and therapeutic or diagnostic modality. Deduplicated project text was further analyzed using MedCPT biomedical embeddings and BERTopic to identify higher-order thematic domains.

**Results:**

The cohort represented approximately USD 343.8 million in cumulative NCI funding in BRDPI-adjusted 2025 U.S. dollars. Annual research activity increased over time, peaking in 2015 with 53 active AYs and approximately USD 24.8 million in BRDPI-adjusted 2025 U.S. dollars. Translational or mixed clinical–preclinical research accounted for the largest share of the portfolio (46.5%), and R01 awards were the dominant funding mechanism (48.5%). Papillary thyroid carcinoma was the most frequently annotated subtype, followed by anaplastic, differentiated, follicular, poorly differentiated and medullary thyroid cancers. BRAF, RET, MAPK/MEK, PI3K/AKT/mTOR and RAS were the leading molecular targets. Topic modelling identified five higher-order thematic domains, suggesting a relative shift from broad thyroid cancer characterization toward diagnosis and risk stratification, molecular signaling, treatment-response biology and therapeutics for aggressive or advanced disease.

**Conclusions:**

NCI-administered thyroid cancer research funding expanded between 1996 and 2025 and became increasingly translational, molecularly informed and clinically oriented. These findings provide an empirical map of public research investment in thyroid oncology and may help inform future funding priorities.

## 1 Introduction

Thyroid cancer is the most common malignancy of the endocrine system and ranks among the fastest-growing cancer diagnoses worldwide. Global Burden of Disease analyses indicate that age-standardized incidence has risen persistently since 1990, reaching an estimated 2 million prevalent cases and approximately 44,800 deaths globally in 2021; projection models anticipate a further 30% increase in incident cases by 2030 (1–3). In the United States, approximately 44,000 new cases are diagnosed annually, and 5-year relative survival exceeds 98% for differentiated tumours, yet clinically important heterogeneity across histologic subtypes and risk strata continues to demand refined diagnostic and therapeutic strategies (4–6).

The molecular landscape of thyroid cancer has become progressively better resolved. Alterations involving BRAF, RET, RAS, TERT promoter and PI3K/AKT/mTOR pathways now anchor current pathogenic models and increasingly inform risk stratification, systemic therapy selection and redifferentiation approaches (4, 7–10). This molecular framework has catalysed major therapeutic developments, including dabrafenib plus trametinib for BRAF V600E-mutant anaplastic thyroid carcinoma (ATC) and BRAF-directed strategies in radioactive iodine-refractory differentiated thyroid cancer (11, 12); selpercatinib for RET-mutant medullary thyroid carcinoma (MTC) and RET fusion-positive differentiated thyroid cancer, with the phase 3 LIBRETTO-531 trial establishing superiority over prior standard of care (13–15); emerging lenvatinib-plus-pembrolizumab combination approaches for ATC/PDTC (16); and MAPK-directed redifferentiation protocols to restore radioactive iodine (RAI) avidity in RAI-refractory differentiated thyroid cancer (RAIR-DTC) (17, 18). The 2025 American Thyroid Association guideline on differentiated thyroid cancer further underscores the growing role of molecular profiling in risk stratification and management decisions (10).

Within this rapidly evolving scientific and clinical landscape, the National Cancer Institute (NCI) represents a central public-investment pillar for thyroid cancer research in the United States. Its portfolio spans the full research continuum, including basic tumour biology, molecular mechanism discovery, diagnostic innovation, translational model systems, early-phase therapeutic development and investigator-initiated clinical research (19). Analysing this portfolio therefore offers an opportunity to examine how public research support has evolved alongside major changes in thyroid cancer epidemiology, molecular classification and precision oncology. Aggregate funding totals alone, however, cannot resolve the internal structure of this investment. Public disease-category estimates, such as those generated through the NIH Research, Condition, and Disease Categorization (RCDC) system, are useful for broad reporting but do not distinguish whether funding is concentrated in specific tumour subtypes, molecular targets, therapeutic or diagnostic modalities, research stages, institutions or thematic domains. A project-level portfolio analysis focused on NCI-administered projects whose primary scientific aim targets thyroid cancer can therefore provide a more precise view of the field’s public research backbone: not only how much was funded, but which research stages, institutions, disease subtypes, molecular targets and thematic areas accounted for sustained or increasing shares of NCI-supported thyroid cancer research over time.

To characterize this internal structure, quantitative funding analysis can be complemented by semantic analysis of project-level text. Rule-based annotation can capture predefined dimensions such as research stage, tumour subtype, molecular target, and therapeutic or diagnostic modality, while topic modelling can identify latent thematic patterns across project titles and abstracts. BERTopic, which integrates transformer-based document embeddings, dimensionality reduction, density-based clustering, and class-based TF-IDF, generates interpretable topics without requiring the number of clusters to be specified in advance (20). Coupled with MedCPT, a biomedical text encoder trained on large-scale PubMed search behaviour, this approach allows NIH project narratives to be analysed with greater semantic fidelity than keyword-based summaries alone (21, 22).

We therefore performed a longitudinal secondary analysis of the NCI thyroid cancer research portfolio over fiscal years 1996–2025. Framed around four interconnected dimensions of the field’s public research backbone, this study examined how funding volume and active project-award year (AY) counts evolved over time; how research stages, funding mechanisms and institutional investment intensity were distributed; how tumour subtypes, molecular targets and therapeutic or diagnostic modalities were emphasized across the portfolio; and how deep-learning-based semantic representations of project narratives revealed higher-order thematic domains and shifts in their relative prominence across three decades. By integrating quantitative funding analysis, rule-based content annotation and BERTopic modelling with MedCPT-derived biomedical embeddings, we aimed to generate a longitudinal map of the scale, structure, scientific emphasis and thematic evolution of NCI-supported thyroid cancer research.

## 2 Materials and methods

### 2.1 Data source and study design

This study is a secondary analysis of publicly available NIH-funded research project records. Project-level grant data were obtained from the NIH Research Portfolio Online Reporting Tools Expenditures and Results (RePORTER) database, the central repository for federally funded biomedical research projects. Retrieved records included project titles, abstracts, project terms, principal investigators, performing institutions, activity codes, fiscal years, funding amounts and administrative institute assignments. We restricted the analytic scope to projects administered by NCI (Administering IC = NCI) because the present study was designed to characterize the NCI public-investment backbone in thyroid cancer research.

### 2.2 Cohort construction and unit of analysis

**Figure 1:**
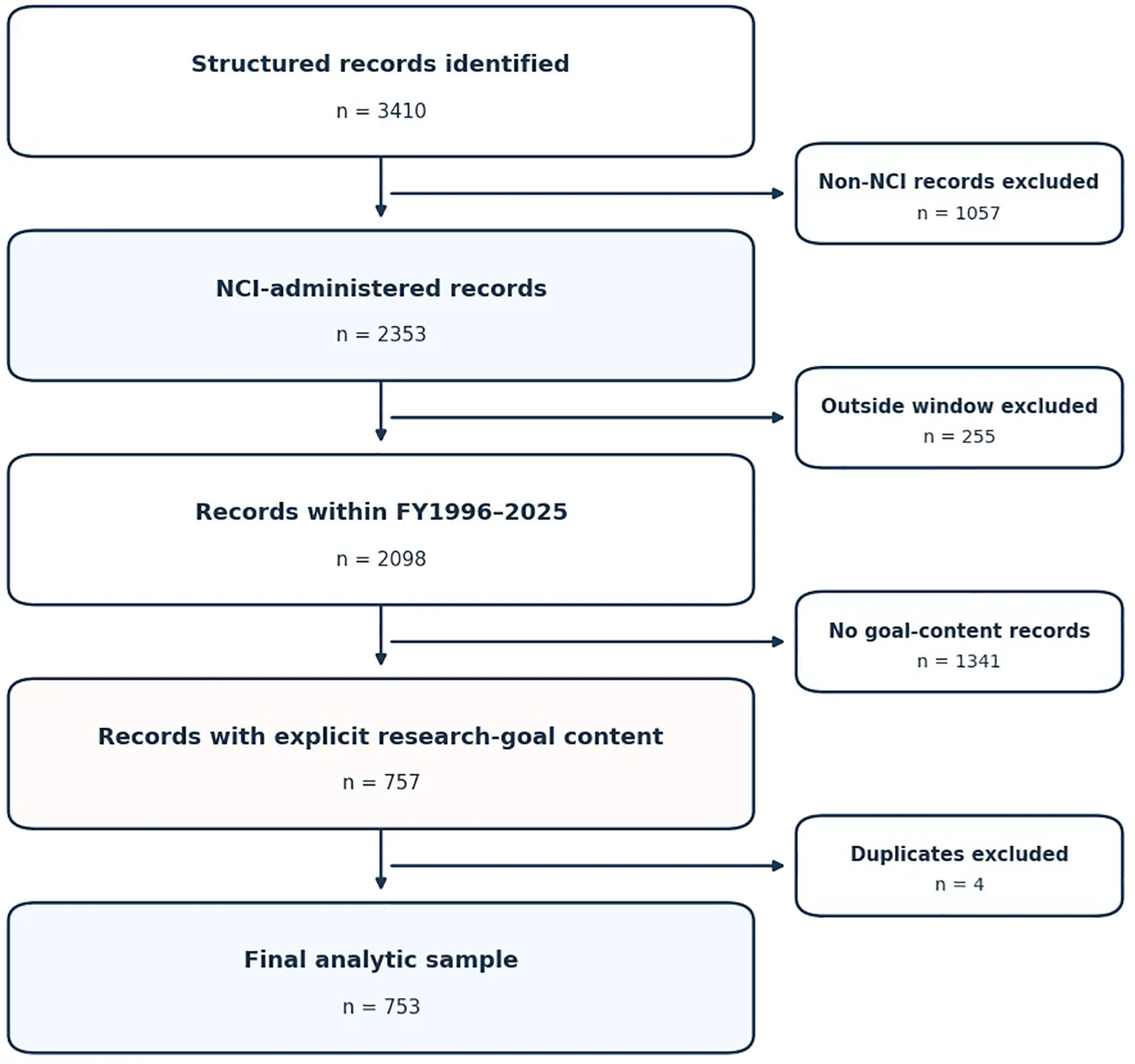
Screening and cohort construction for the NCI thyroid cancer portfolio. Flow diagram showing the sequential construction of the analytic cohort from NIH RePORTER records. Of 3,410 structured records initially identified, 2,353 NCI-administered records were retained after exclusion of 1,057 non-NCI records. Restriction to fiscal years 1996–2025 yielded 2,098 records. Relevance adjudication excluded 1,341 records without explicit thyroid-cancer research-goal content, leaving 757 records. After exclusion of four duplicate records based on Normalized Application ID, the final analytic sample comprised 753 project-award years.

The cohort construction flow is shown in **Figure 1**. Of 3,410 initial RePORTER records, 2,353 were retained after restricting to NCI-administered projects, 2,098 after restricting to fiscal years 1996–2025, 757 after relevance adjudication, and 753 after deduplication on Normalized Application ID.

Although the initial retrieval used the query term “thyroid cancer,” many raw records contained only incidental or peripheral mentions of thyroid cancer. We therefore performed a two-reviewer relevance adjudication process to identify records with explicit thyroid-cancer research-goal content. Two reviewers independently screened candidate records for relevance, with excellent inter-reviewer agreement (Cohen’s kappa = 0.996); discrepant or borderline records were adjudicated by a third reviewer.

### 2.3 BRDPI-based inflation adjustment

Funding amounts were adjusted for inflation using the Biomedical Research and Development Price Index (BRDPI). Annual BRDPI values were obtained from the NIH Office of Budget BRDPI table, and nominal funding amounts for each fiscal year were converted to constant FY2025 U.S. dollars using the ratio of the FY2025 projected BRDPI value to the BRDPI value for the corresponding fiscal year. For funding analyses, subproject/component rows were excluded to minimize potential double counting, whereas award-year counts were calculated using the full analytic set.

### 2.4 Rule-based content annotation

Each project-award year (AY) record was annotated using predefined keyword-based rule sets across four conceptual axes: research stage, disease subtype, molecular target and therapeutic/diagnostic modality. For each record, project title, abstract, public health relevance statement, and project terms were combined into a single searchable text field. Dictionaries were developed to reflect commonly used thyroid cancer terminology, including tumour subtypes, recurrent molecular alterations and major therapeutic or diagnostic modalities described in contemporary thyroid cancer literature and guidelines (4, 7, 20). Research stage was assigned as a mutually exclusive primary category for each award-year record. In contrast, disease subtype, molecular target, and therapeutic/diagnostic modality were annotated as multi-label variables; therefore, a single award-year record could receive more than one subtype, target, or modality label when multiple relevant concepts were identified in the project title, abstract, public health relevance statement, or NIH RePORTER terms.The complete keyword dictionaries and rule-based mapping criteria for the main annotation variables are provided in **Supplementary Table 1**.

### 2.5 BERTopic topic modelling

BERTopic was applied to the deduplicated corpus using MedCPT-derived biomedical text embeddings. BERTopic has been increasingly used for biomedical and health-related text mining applications, including analyses of opioid-related cardiovascular risk narratives, cancer immunotherapy-related online discussions, and hospital patient-safety event narratives (20, 23, 24).

Text embeddings were generated using the MedCPT Article Encoder. The BERTopic workflow included biomedical semantic representation, UMAP dimensionality reduction, HDBSCAN density-based clustering, and class-based TF-IDF keyword extraction (20, 23). Dimensionality reduction was performed using UMAP with n_neighbors = 15, n_components = 5, min_dist = 0.0, metric = “cosine”, and random_state = 42. Clustering was performed using HDBSCAN with min_cluster_size = 20, min_samples = 5, metric = “euclidean”, cluster_selection_method = “eom”, and prediction_data = True. Topic representations were generated using CountVectorizer with English stop words, ngram_range = (1, 2), min_df = 3, and max_df = 0.85, followed by c-TF-IDF weighting.

Documents not assigned to a substantive HDBSCAN cluster were retained as the outlier topic, Topic −1. This procedure yielded eight fine-grained substantive topics and 184 outlier documents, corresponding to 31.6% of the modellable corpus. The eight fine-grained substantive topics were subsequently reviewed and consolidated into five higher-order thematic domains based on topic keywords, representative documents, and thematic coherence. Fine-grained BERTopic topics were reviewed by the investigators and consolidated into higher-order thematic domains based on top c-TF-IDF keywords, representative project documents, C_v topic coherence scores, semantic overlap across topics, and portfolio-level interpretability (**Supplementary Table 2**). The consolidated domains were used as descriptive summaries of related fine-grained topics rather than as independently generated model outputs.

### 2.6 Descriptive analysis and visualization

All data processing, aggregation and figure generation were performed in Python. Analyses were descriptive. Continuous variables, including funding amounts and project counts, were summarized as totals or annual aggregates. Categorical variables were summarized as counts and percentages. Temporal trends, funding mechanisms, institutional distribution, molecular-target trajectories and BERTopic-derived visualizations were generated for manuscript and supplementary reporting.

## 3 Results

### 3.1 Cohort characteristics

The final analytic cohort comprised 753 project-award years (AYs), corresponding to 156 unique core NCI-administered thyroid cancer research projects, with cumulative nominal funding of approximately USD 283.8 million over fiscal years 1996–2025. Because all retained records were NCI-administered, the present analysis characterizes the internal structure of the NCI thyroid cancer portfolio rather than cross-institute differences. Overall characteristics are summarized in **Table 1**. Translational/mixed clinical–preclinical work represented the largest research-stage category (350 AYs; 46.5%), followed by preclinical (190; 25.2%), clinical (182; 24.2%) and unspecified/basic work (31; 4.1%). R01 grants represented the largest funding-mechanism category, accounting for 365 AYs (48.5%), followed by Program/Center grants (97 AYs; 12.9%), Training/Career awards (92 AYs; 12.2%) and Intramural projects (73 AYs; 9.7%).

**Table 1:** Overall characteristics of the NCI thyroid cancer research portfolio, 1996–2025.

Indicator	Value
Project-award years (AYs; primary analytic unit)	753
Unique core projects	156
Cumulative funding, 2025 USD, BRDPI-adjusted	$343.805 million
Clinical AYs	182 (24.2%)
Translational/mixed clinical–preclinical AYs	350 (46.5%)
Preclinical AYs	190 (25.2%)
Unspecified/basic AYs	31 (4.1%)
R01 AYs	365 (48.5%)
Program/Center (P) AYs	97 (12.9%)
Training/Career (F/K) AYs	92 (12.2%)
Intramural (Z) AYs	73 (9.7%)

AY, project-award year. Percentages in the lower block refer to the AY denominator (n = 753). R01 unique-project percentage computed from 156 core projects.

### 3.2 Temporal evolution of funding and research stage

**Figure 2:**
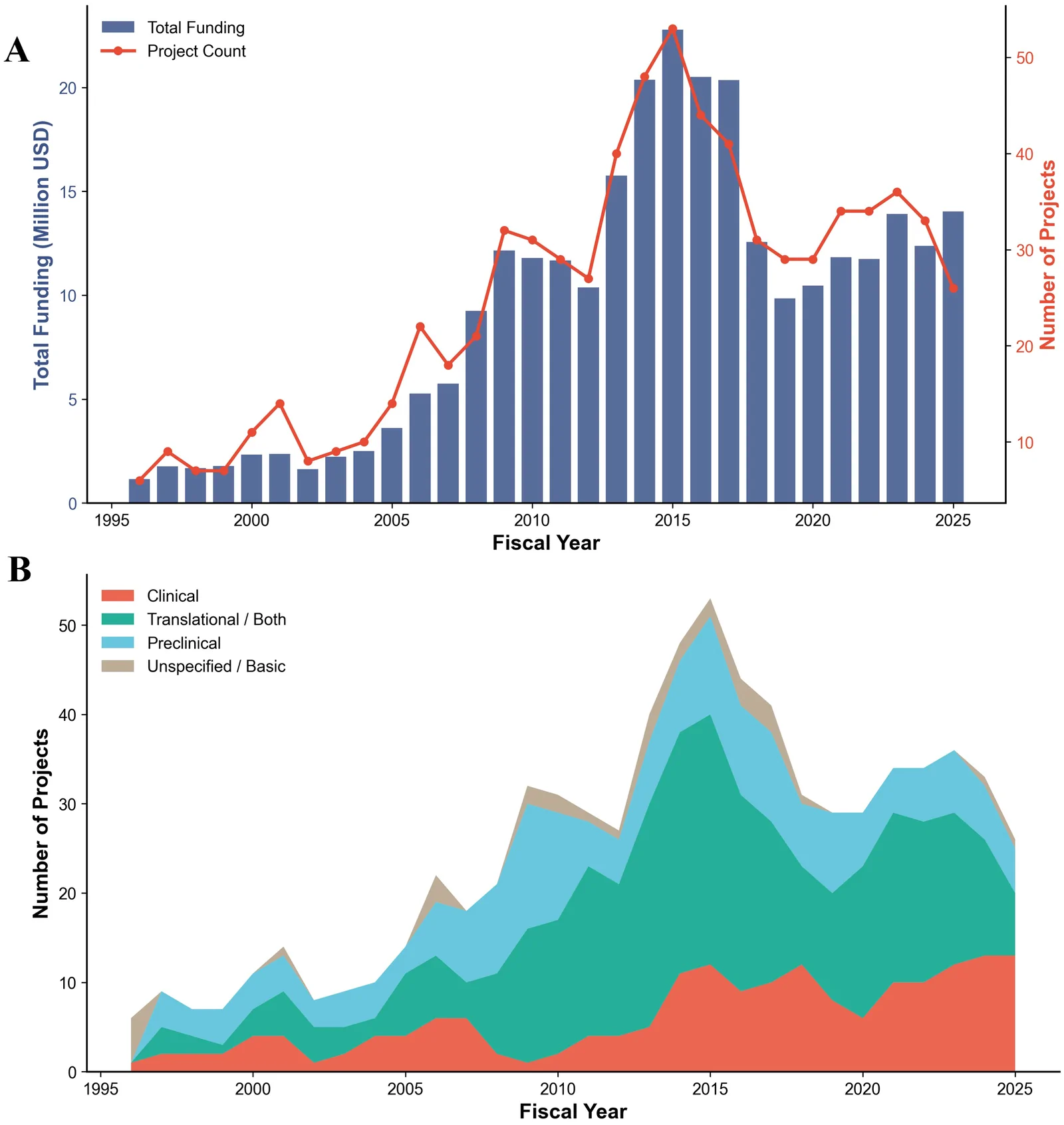
Temporal trends in NCI thyroid cancer funding and research-stage composition. **(A)** Annual BRDPI-adjusted funding and active project-award year (AY) counts. Blue bars show total annual funding in million 2025 U.S. dollars after adjustment using the Biomedical Research and Development Price Index (BRDPI), and the red line shows the number of active AY records in each fiscal year. **(B)** Annual research-stage composition of active AY records, classified as clinical, translational/mixed, preclinical or unspecified/basic.

Annual active AY counts and BRDPI-adjusted funding increased overall across the study window (**Figure 2A**). In 1996, six AYs were active. Funding increased after the mid-2000s and reached a sustained high-activity period between 2014 and 2017. The peak year was 2015, with 53 active AYs and approximately USD 24.8 million in BRDPI-adjusted 2025 constant U.S. dollars. Research-stage composition shifted over the same interval (**Figure 2B**).

Preclinical and unspecified/basic work accounted for a larger share of active AYs in the late 1990s and early 2000s, whereas translational/mixed clinical–preclinical work increased after 2010 and became the leading category in the subsequent decade. Clinical AYs also accounted for a larger share after 2020. Together, these patterns indicate a portfolio-level shift from earlier mechanism-oriented and preclinical work toward more translational and clinically oriented research.

### 3.3 Funding mechanisms and institutional distribution

**Figure 3:**
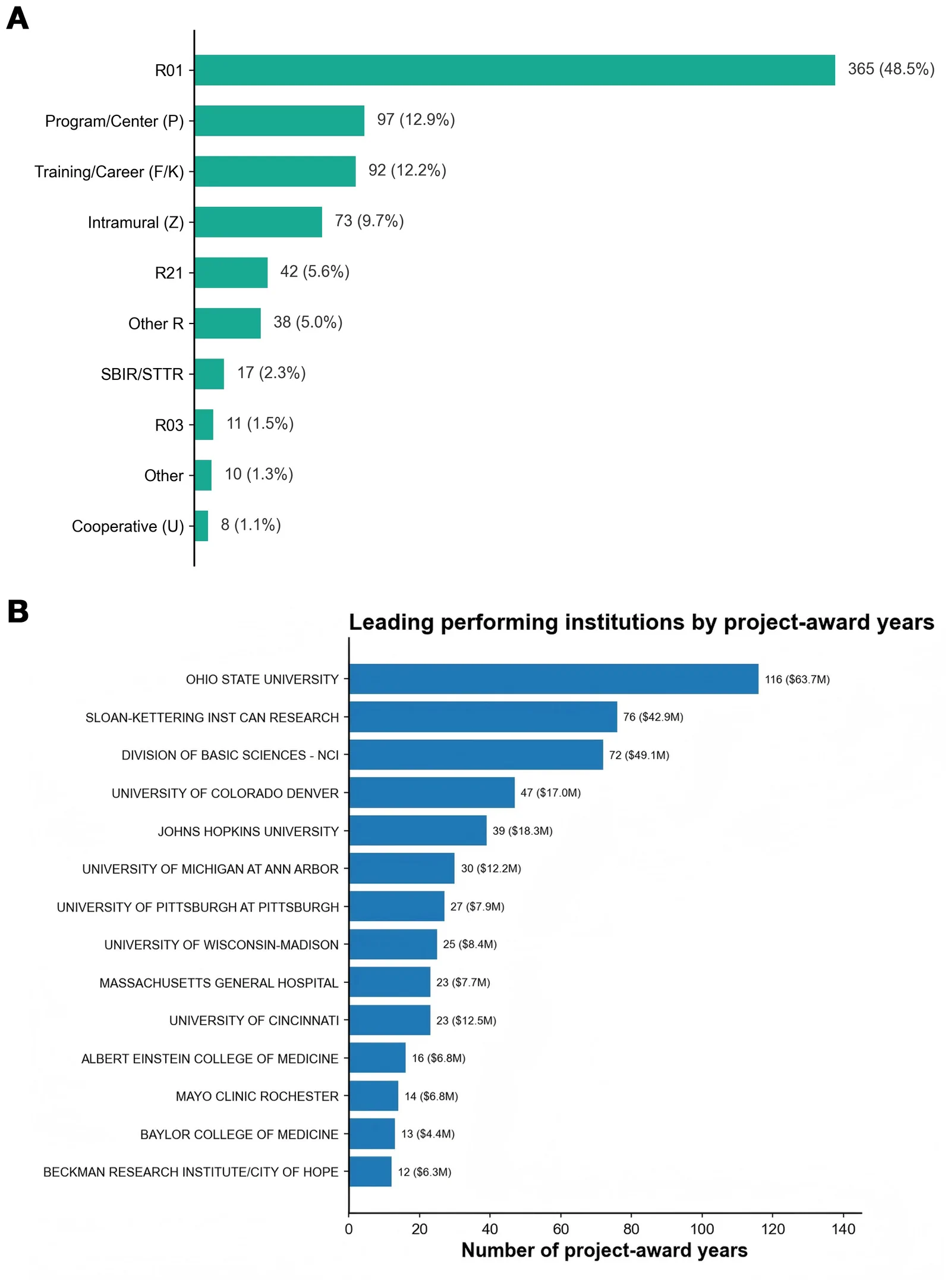
Funding mechanisms and institutional distribution of NCI thyroid cancer research. **(A)** Distribution of project-award years by funding mechanism. Bar labels show AY counts and percentages of the final analytic cohort. **(B)** Leading performing institutions ranked by AY count. Labels show AY counts with cumulative BRDPI-adjusted 2025 funding in parentheses.

Funding mechanisms were dominated by R01 grants, which accounted for 365 AYs (48.5%), followed by Program/Center grants (97 AYs; 12.9%), Training/Career awards (92 AYs; 12.2%) and Intramural projects (73 AYs; 9.7%) (**Figure 3A**). Smaller contributions came from R21 awards (42 AYs; 5.6%), other R-series mechanisms (38 AYs; 5.0%), SBIR/STTR awards (17 AYs; 2.3%), R03 awards (11 AYs; 1.5%), other mechanisms (10 AYs; 1.3%) and Cooperative agreements (8 AYs; 1.1%). Institutional support was concentrated among a small number of high-output centres (**Figure 3B**; **Table 2**). Among extramural institutions, Ohio State University led by AY count and cumulative BRDPI-adjusted 2025 funding (116 AYs; USD 63.7 million), followed by the Sloan-Kettering Institute for Cancer Research (76 AYs; USD 42.9 million), the University of Colorado Denver (47 AYs; USD 17.0 million) and Johns Hopkins University (39 AYs; USD 18.3 million). NCI intramural research contributed 73 AYs and USD 49.06 million. Because intramural NCI research is funded through direct appropriation rather than competitive extramural grant mechanisms, it is reported separately from extramural institutions in **Table 2**.

**Table 2:** Leading performing institutions by project-award years.

Institution	Type	AYs	Unique projects	Avg AYs/ proj	Funding, 2025 USD M
OHIO STATE UNIVERSITY	Extramural	116	16	7.2	63.68
SLOAN-KETTERING INST CAN RESEARCH	Extramural	76	13	5.8	42.87
UNIVERSITY OF COLORADO DENVER	Extramural	47	13	3.6	16.98
JOHNS HOPKINS UNIVERSITY	Extramural	39	11	3.5	18.28
UNIVERSITY OF MICHIGAN AT ANN ARBOR	Extramural	30	8	3.8	12.20
UNIVERSITY OF PITTSBURGH AT PITTSBURGH	Extramural	27	4	6.8	7.90
UNIVERSITY OF WISCONSIN-MADISON	Extramural	25	4	6.2	8.38
UNIVERSITY OF CINCINNATI	Extramural	23	4	5.8	12.55
NCI	Intramural	73	13	5.6	49.06

### 3.4 Disease subtypes, molecular targets and therapeutic/diagnostic modalities

**Figure 4:**
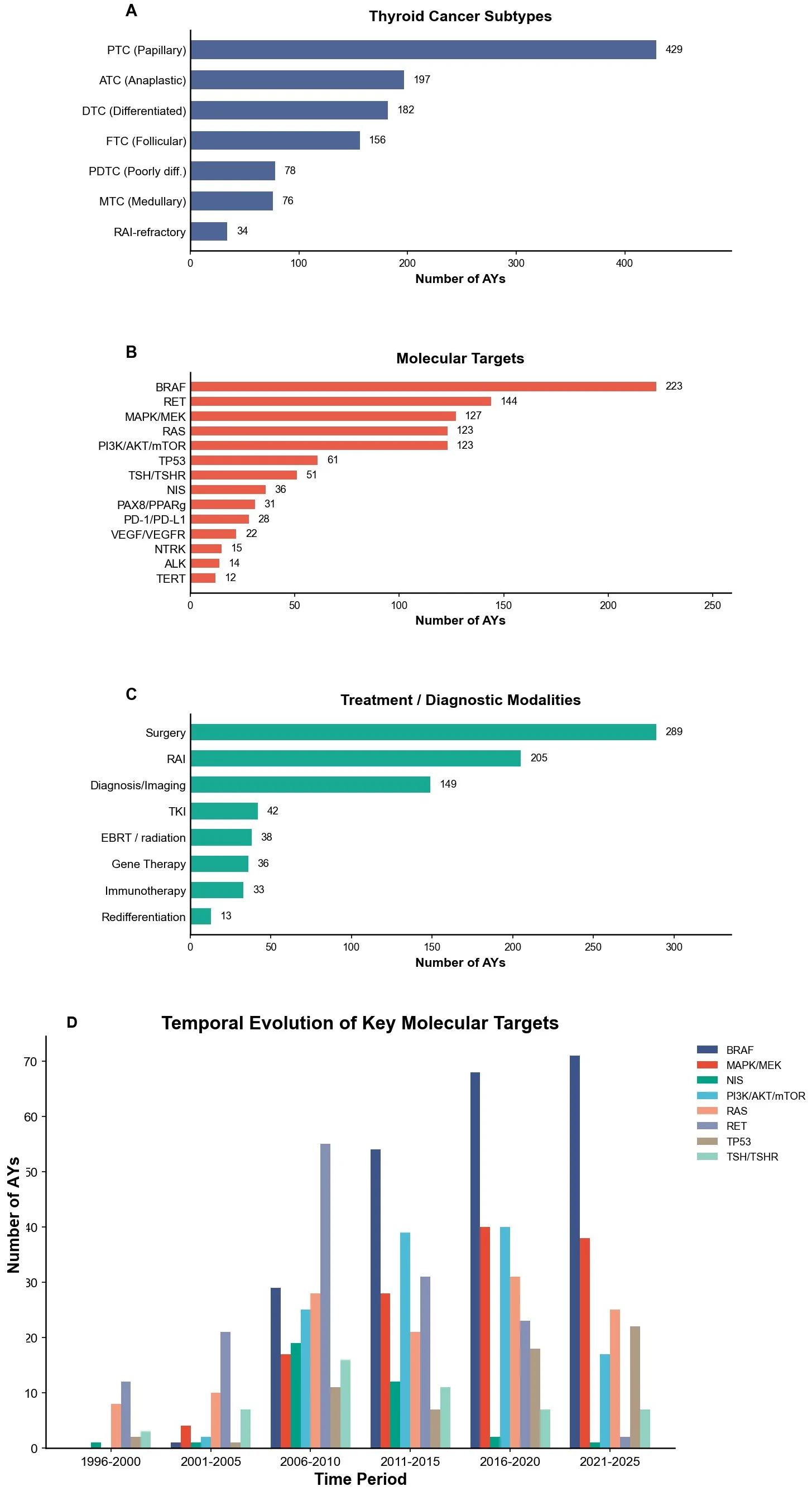
Disease subtype, molecular target and therapeutic/diagnostic modality landscape of the NCI thyroid cancer portfolio. **(A)** Overall project-award year (AY) counts for annotated thyroid cancer subtypes. **(B)** Overall AY counts for annotated molecular targets. **(C)** Overall AY counts for annotated therapeutic and diagnostic modalities. **(D)** Five-year temporal distribution of key molecular target annotations. Bar heights indicate AY counts within each five-year period.

Among annotated disease subtypes (**Figure 4A**), PTC was the most frequently studied (429 AYs), reflecting both its epidemiological dominance and its role as the canonical model for thyroid cancer molecular biology. ATC (197 AYs), DTC (182), FTC (156), PDTC (78), and MTC (76) followed. The disproportionate attention to ATC relative to its clinical incidence (~1%–2% of all thyroid cancers) reflects sustained scientific interest in this lethal undifferentiated subtype and the therapeutic urgency created by its dismal prognosis (4, 24).

BRAF (223 AYs) dominated the molecular-target landscape, followed by RET (144), MAPK/MEK (127), PI3K/AKT/mTOR (123), and RAS (123), with smaller contributions from TP53, NIS, TSH/TSHR, PAX8/PPARγ, PD-1/PD-L1, TERT, NTRK and ALK (**Figure 4B**). This distribution reflects the established roles of these alterations as drivers of thyroid tumorigenesis, prognostic markers and clinically actionable targets (4, 7–10). In therapeutic and diagnostic modalities (**Figure 4C**), surgery (289 AYs), RAI (205), and imaging/diagnostics (149) predominated, with TKI, immunotherapy, redifferentiation therapy and gene therapy constituting growth areas in later years. Temporal analysis (**Figure 4D**) showed that RET-focused work was prominent in earlier and middle windows (2006–2010 peak), whereas BRAF-centred activity rose after 2011 and has dominated since 2016 — a trajectory concordant with the clinical emergence of BRAF/MEK-directed therapies and redifferentiation protocols (11, 12, 17–19). MAPK/MEK and PI3K/AKT/mTOR investment expanded in parallel after 2011, consistent with growing recognition of signalling network complexity in thyroid cancer resistance and progression.

### 3.5 BERTopic higher-order thematic domains and temporal evolution

**Figure 5:**
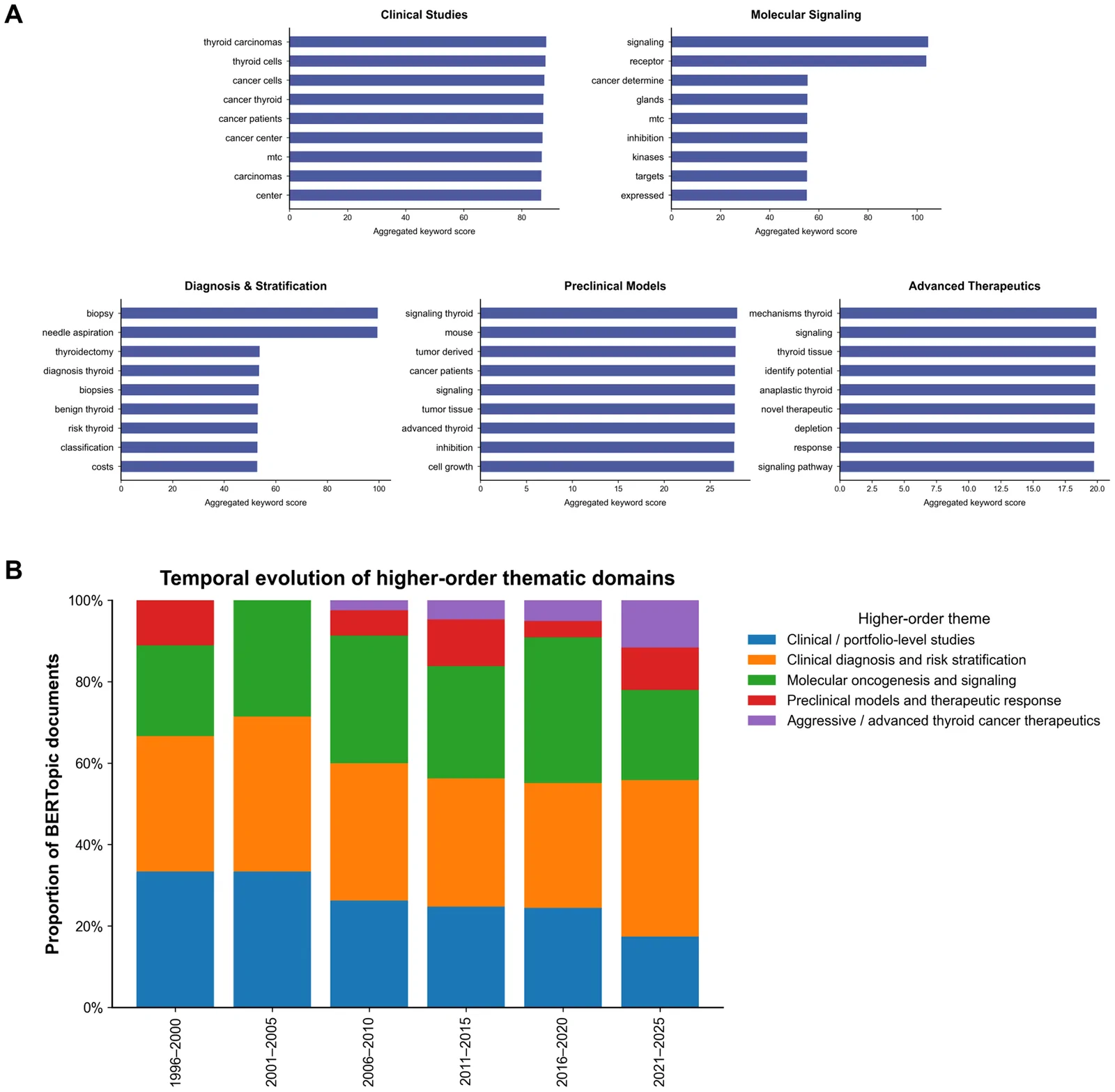
BERTopic keyword signatures and temporal evolution of higher-order thematic domains. **(A)** Representative keyword signatures for the five investigator-consolidated higher-order thematic domains derived from BERTopic fine-grained topics. Bar lengths indicate aggregated keyword scores within each domain. **(B)** Temporal evolution of higher-order thematic domains across six five-year periods from 1996 to 2025. Stacked bars show the proportion of BERTopic-modellable documents assigned to each domain in each period.

BERTopic modelling identified five investigator-consolidated higher-order thematic domains within the NCI thyroid cancer portfolio (**Figure 5**): clinical/portfolio-level thyroid cancer characterization, clinical diagnosis and risk stratification, molecular oncogenesis and signaling, preclinical models and therapeutic response, and aggressive/advanced thyroid cancer therapeutics.

The keyword signatures of these domains reflected distinct areas of portfolio content (**Figure 5A**). Clinical/portfolio-level characterization captured broad thyroid cancer, tumour-cell and portfolio-level terminology. Clinical diagnosis and risk stratification was characterized by biopsy, thyroidectomy, classification and risk-related terms. Molecular oncogenesis and signaling was defined by receptor, kinase, signalling and pathway-related terminology. Preclinical models and therapeutic response reflected mouse models, tumour-derived systems, inhibition and treatment-response biology. Aggressive/advanced thyroid cancer therapeutics was characterized by advanced thyroid cancer, anaplastic thyroid cancer, novel therapeutic strategies and response-related terms. The temporal evolution of these domains is shown in **Figure 5B**.

## 4 Discussion

This longitudinal portfolio analysis characterized nearly three decades of NCI-supported thyroid cancer research across funding volume, portfolio structure, disease and molecular emphasis, and thematic evolution. Overall, the findings suggest that NCI-supported thyroid cancer research transitioned from a relatively small and episodic portfolio in the late 1990s and early 2000s into a sustained and more established research programme by the mid-2010s. The post-2006 expansion and subsequent stabilization of project-award-year counts and nominal funding suggest not only increased investment, but also the consolidation of thyroid cancer as a durable area of NCI-supported oncology research. This trajectory is consistent with the growing clinical and public-health salience of thyroid cancer over the same period. Although rising diagnosed incidence partly reflects increased detection and overdiagnosis, the expanding diagnosed population has increased the need for risk stratification, long-term surveillance and treatment decision-making (2, 25, 26). In parallel, advances in molecularly guided therapy for radioiodine-refractory, metastatic and aggressive thyroid cancers have strengthened the translational relevance of thyroid cancer research (27, 28).

Placing NCI thyroid cancer funding patterns in the context of disease burden provides additional policy relevance. In our analysis, both active award-year counts and BRDPI-adjusted funding peaked around 2015 and declined thereafter. This decline should be interpreted against thyroid cancer’s continued contribution to cancer incidence, survivorship care, and long-term clinical burden. Boucai, Zafereo, and Cabanillas estimated that approximately 43,720 new thyroid carcinoma cases were expected in the United States in 2023 and reported high overall 5-year relative survival, underscoring the need for risk-adapted follow-up care (4). Prior work by Aschebrook-Kilfoy et al. showed that sustained increases in thyroid cancer incidence have important clinical and economic consequences and that thyroid cancer has been underfunded relative to its frequency among cancers (29). Zhao et al. further demonstrated that thyroid cancer burden is reflected not only in incidence and mortality, but also in disability-adjusted life years (30). Together, these studies suggest that thyroid cancer has a multidimensional disease burden that may not be fully captured by mortality alone.

This post-2015 decline in NCI thyroid cancer funding observed in our study suggests that research support has not increased in parallel with thyroid cancer’s continued incidence, survivorship needs, and long-term clinical burden. These findings support reassessing thyroid cancer’s position within NCI research funding priorities and suggest that renewed or expanded investment may be warranted, particularly for aggressive, advanced, metastatic, and radioiodine-refractory thyroid cancer, where outcomes remain poorer and therapeutic needs remain incompletely met. This interpretation is consistent with broader analyses showing that NIH and global cancer research investments may not always align closely with disease burden or policy need, as well as recent discussion of NIH support for the thyroid field (31–33).

In addition to disease-burden considerations, the post-2015 decline may also reflect broader portfolio dynamics rather than an overall contraction in federal biomedical research funding, as total NIH and NCI appropriations increased in nominal terms after 2015. The emergence of cross-cutting initiatives such as the Cancer Moonshot may have supported some thyroid cancer–relevant work through broader pan-cancer or mechanism-based programs not captured by our thyroid cancer–specific RePORTER criteria. Other possible contributors, including changes in investigator workforce, application volume, grant success rates, or NCI portfolio priorities, could not be directly assessed in the present descriptive analysis.

The portfolio also showed evidence of increasing translational and clinically oriented activity. Translational and mixed clinical–preclinical research constituted the largest research-stage category, suggesting that NCI-supported thyroid cancer research was concentrated at the interface between molecular discovery and clinical application. Within the text-modellable corpus, the relative decline of the clinical/portfolio-level characterization domain further suggests that the portfolio moved beyond broad clinical description toward more specialized molecular, diagnostic and therapeutic themes. Funding-mechanism patterns were consistent with this maturation. R01 awards remained the dominant mechanism overall, indicating that independent research project grants formed the central backbone of the portfolio. This finding is consistent with prior NCI workforce analysis showing that R01-funded investigators constitute a major component of the cancer research enterprise, while also highlighting broader challenges related to workforce aging, delayed entry into first R01 funding and early-career retention (34). Program/Center awards, Training/Career mechanisms and NCI intramural projects contributed smaller but meaningful shares, pointing to a complementary funding architecture aligned with multi-project and center-based research, workforce and career development, and intramural research capacity.

The disease-subtype and molecular-target landscape was consistent with the maturation of thyroid precision oncology. PTC was the most frequently annotated subtype, reflecting its epidemiological predominance, whereas ATC received substantial attention despite its lower incidence, consistent with its aggressive clinical course, poor prognosis and unmet therapeutic need (4, 35). At the molecular level, BRAF, RET, MAPK/MEK, PI3K/AKT/mTOR and RAS accounted for the largest shares of target annotations. The rise of BRAF-centred activity after 2011, together with sustained RET and MAPK/MEK representation, was temporally aligned with major therapeutic developments in BRAF/MEK-targeted therapy, selective RET inhibition and MAPK-directed redifferentiation strategies (7, 11, 13, 17).

The temporal evolution of higher-order BERTopic domains provides a more integrated view of this maturation. Early portfolio text was more heavily weighted toward clinical and portfolio-level characterization, whereas this domain declined progressively over time, suggesting that NCI-supported thyroid cancer research moved beyond broad disease description as the portfolio matured. In contrast, clinical diagnosis and risk stratification remained a persistent theme across the study period, consistent with ongoing efforts to distinguish indolent from clinically significant disease and to refine biopsy, thyroidectomy and surveillance decisions (10). Molecular oncogenesis and signaling also remained a major recurrent domain, reflecting the central role of BRAF-, RET-, RAS- and PI3K/AKT/mTOR-related alterations in thyroid cancer pathogenesis, progression and molecularly informed prognostication (36). The aggressive thyroid cancer therapeutics domain expanded in the most recent period, aligning with the growing therapeutic relevance of BRAF/MEK inhibition, selective RET inhibition and MAPK-directed redifferentiation strategies aimed at restoring RAI avidity in advanced or radioiodine-refractory disease. Together, these temporal patterns suggest that the semantic evolution of the NCI thyroid cancer portfolio was not merely a shift toward molecular research, but a broader rebalancing toward risk-adapted diagnosis, molecularly informed disease stratification and therapeutically actionable advanced-disease research.

## 5 Conclusions

This longitudinal portfolio analysis shows that NCI-supported thyroid cancer research expanded substantially between 1996 and 2025 and matured toward a more translational, molecularly informed and clinically oriented research programme. The portfolio retained a strong R01 foundation while also incorporating centre-level, training/career and intramural mechanisms, with extramural support concentrated among several high-output institutions. Disease, target and modality annotations highlighted sustained emphasis on PTC, ATC, BRAF, RET, MAPK/MEK, PI3K/AKT/mTOR, RAS, surgery, RAI and imaging/diagnostics. Topic modelling further identified a shift from broad characterization toward diagnosis and risk stratification, molecular signaling, treatment-response biology and targeted therapeutics for aggressive or advanced thyroid cancer. These findings provide a structured empirical map of the NCI thyroid cancer research portfolio and may help calibrate future public investment against the evolving scientific and clinical landscape of thyroid oncology.

## 6 Limitations

This study has several limitations. First, the analysis was limited to NCI-administered projects captured in NIH RePORTER and therefore does not represent the full thyroid cancer research funding ecosystem, including other NIH institutes, non-NCI federal agencies, foundations, industry, philanthropic organizations, or international funders. Second, some relevant projects may have been missed if thyroid cancer was not explicitly described in the project title, abstract, public health relevance statement, or NIH RePORTER terms; conversely, award amounts may not perfectly reflect thyroid cancer–specific investment when projects addressed multiple cancer types or broader biological mechanisms. Third, although BRDPI-adjusted funding analyses were added to improve comparability across time, long-term funding trends may still be influenced by changes in research costs, grant mechanisms, institutional indirect costs, and NIH reporting practices. Fourth, rule-based annotation may introduce misclassification, particularly for projects with broad or ambiguous terminology. Finally, BERTopic-derived themes should be interpreted as descriptive semantic patterns rather than exhaustive taxonomic categories because topic assignments depend on available project text, model parameters, investigator consolidation, and the substantial proportion of Topic −1 documents.

## Data Availability

Publicly available datasets were analyzed in this study. Underlying project records are publicly available via NIH RePORTER (https://reporter.nih.gov). Derived analytic files are available from the corresponding author upon reasonable request.
