# Supplementary material for: Thirty years of NCI thyroid cancer research investment (1996–2025): a longitudinal portfolio analysis of funding trends, molecular target emphasis and thematic evolution
Source: Front Oncol. 2026 Aug 17;16:1876183. doi: 10.3389/fonc.2026.1876183 (PMC13526602; doi:10.3389/fonc.2026.1876183)
Supplement: Supplementary file 1 [file Table1.docx]

**Supplementary Table S1. Mapping of non-outlier BERTopic topics to higher-order thematic domains**

| **Final higher-order thematic domain** | **Original BERTopic topics merged** | **Fine-grained topic labels** | **C_v coherence scores** |
| --- | --- | --- | --- |
| Clinical, registry, and portfolio-level studies | Topic 0 | Clinical thyroid cancer / registry-oriented studies | 0.302 |
| Molecular oncogenesis and signaling | Topic 1; Topic 3 | Oncogenic signaling pathways; BRAF / tumor biology / anaplastic thyroid | 0.318; 0.470 |
| Clinical diagnosis and risk stratification | Topic 2; Topic 4; Topic 6 | Thyroid nodule diagnosis / thyroidectomy; Thyroid neoplasms / molecular markers; Diagnosis / stratification / screening | 0.743; 0.335; 0.319 |
| Preclinical models and therapeutic response | Topic 5 | Mouse / preclinical / tumor-derived models | 0.448 |
| Aggressive / advanced thyroid cancer therapeutics | Topic 7 | Anaplastic thyroid / novel therapeutics | 0.468 |

Abbreviations: BERTopic, Bidirectional Encoder Representations from Transformers topic modeling; C_v, topic coherence score.
