# Supplementary material for: Thirty years of NCI thyroid cancer research investment (1996–2025): a longitudinal portfolio analysis of funding trends, molecular target emphasis and thematic evolution
Source: Front Oncol. 2026 Aug 17;16:1876183. doi: 10.3389/fonc.2026.1876183 (PMC13526602; doi:10.3389/fonc.2026.1876183)
Supplement: Supplementary file 2 [file Table2.docx]

**Supplementary Table S2. Rule-based annotation dictionaries and mapping rules**

| **Domain** | **Label** | **Keywords / matching patterns** |
| --- | --- | --- |
| Study type | Clinical | clinical trial; phase 1/2/3; patient cohort/sample; patients with; recruit; human subject; randomized; retrospective; observational study; registry; cohort study; case-control; epidemiolog; surveillance; SEER; NCDB |
| Study type | Preclinical | mouse; mice; murine; xenograft; PDX; in vitro; in vivo; cell line; cell culture; preclinical; animal model; tumor model; syngeneic; orthotopic |
| Study type | Translational / Both | clinical keyword set and preclinical keyword set |
| Study type | Unspecified / Basic | no clinical or preclinical keyword hit |
| Thyroid cancer subtype | PTC / Papillary | PTC; papillary; papillary thyroid |
| Thyroid cancer subtype | FTC / Follicular | FTC; follicular thyroid; follicular carcinoma |
| Thyroid cancer subtype | MTC / Medullary | MTC; medullary thyroid; medullary carcinoma |
| Thyroid cancer subtype | ATC / Anaplastic | ATC; anaplastic; undifferentiated thyroid |
| Thyroid cancer subtype | DTC / Differentiated | DTC; differentiated thyroid; well-differentiated |
| Thyroid cancer subtype | PDTC / Poorly differentiated | PDTC; poorly differentiated |
| Thyroid cancer subtype | RAI-refractory | RAI refractory; RAI-refractory; radioiodine refractory; radioiodine-refractory; iodine refractory; iodine-refractory; RAI resistant; RAI-resistant; radioiodine resistant; radioiodine-resistant |
| Molecular target | BRAF | BRAF; V600E |
| Molecular target | RAS | RAS; NRAS; HRAS; KRAS |
| Molecular target | RET | RET; RET/PTC; RET fusion |
| Molecular target | VEGF/VEGFR | VEGF; VEGFR |
| Molecular target | PI3K/AKT/mTOR | PI3K; AKT; mTOR; PIK3CA |
| Molecular target | MAPK/MEK | MAPK; MEK; ERK |
| Molecular target | TP53 | TP53; p53 |
| Molecular target | TERT | TERT; TERT promoter |
| Molecular target | NIS | NIS; sodium-iodide symporter; sodium iodide symporter |
| Molecular target | TSH/TSHR | TSH; TSHR; thyrotropin |
| Molecular target | PAX8/PPARg | PAX8; PPARG; PPARg; PAX8-PPARG; PAX8/PPARG |
| Molecular target | ALK | ALK; ALK fusion |
| Molecular target | NTRK | NTRK; TRK |
| Molecular target | PD-1/PD-L1 | PD-1; PD1; PD-L1; PDL1; immune checkpoint; checkpoint inhibit; checkpoint blockade |
| Treatment / diagnostic modality | RAI / Radioiodine | RAI; radioiodine; I-131; I 131; iodine-131; iodine 131; radioactive iodine |
| Treatment / diagnostic modality | TKI | TKI; tyrosine kinase inhibitor; sorafenib; lenvatinib; cabozantinib; vandetanib; selpercatinib; pralsetinib |
| Treatment / diagnostic modality | Immunotherapy | immunotherapy; immune checkpoint; anti-PD; anti PD; CAR-T; CAR T; pembrolizumab; nivolumab; ipilimumab |
| Treatment / diagnostic modality | Surgery | thyroidectomy; surgical; surgery; lobectomy; lymph node dissection; neck dissection |
| Treatment / diagnostic modality | EBRT / Radiation | external beam; EBRT; radiation therapy; radiotherapy |
| Treatment / diagnostic modality | Redifferentiation | redifferentiat; re-differentiat; iodine uptake restor |
| Treatment / diagnostic modality | Gene therapy | gene therapy; CRISPR; gene editing; siRNA; shRNA; antisense |
| Treatment / diagnostic modality | Diagnosis / Imaging | FNA; fine needle; ultrasound; PET-CT; PET CT; PET; molecular diagnostic; liquid biopsy; Thyrogen |

Abbreviations: ATC, anaplastic thyroid cancer; DTC, differentiated thyroid cancer; EBRT, external beam radiotherapy; FTC, follicular thyroid cancer; MTC, medullary thyroid cancer; NCDB, National Cancer Database; PDTC, poorly differentiated thyroid cancer; PTC, papillary thyroid cancer; RAI, radioactive iodine; SEER, Surveillance, Epidemiology, and End Results Program; TKI, tyrosine kinase inhibitor.
